# Southern Medical College Commencement

**Published:** 1880-03-20

**Authors:** R. C. Word

**Affiliations:** Dean, Atlanta, Ga.


### *SOUTHERN MEDICAL COLLEGE COMMENCEMENT.*

The Commencement Exercises of the SOUTHERN MEDICAL COLLEGE took place on the evening of the 26th of February, 1880, in the Representative Hall, Atlanta, Georgia. There was present a very large and intelligent audience, filling both hall and galleries.

A stage was erected about the Speaker's stand, upon which the Professors were seated, and upon the front seats adjacent were the Board of Trustees, ministers of the city and students of the class. The Hall was brilliantly lighted by the circle of gas jets in the dome, and the scene enlivened by the array of female beauty and attraction.

The Programme for the occasion was well arranged, each separate part being briefly and pertinently announced by Dr. R. C. WORD, Dean of the Faculty—the intervals being occupied by most delightful music, rendered by the Drs. CRENSHAW and Ladies. The following Programme was observed:

1st. Prayer, by Rev. Dr. BOGGS, pastor of the Central Presbyterian Church.

2d. Salutatory, by J. H. LUMPKIN, who delivered a very appropriate, chaste, scholarly and eloquent address.

3d. Address of Dr. THOS. P. DAVIS, Valedictorian of the class. The young man acquitted himself finely.

4th. Presentation of Diplomas to the Graduates of the class, by Prof. THOS. S. POWELL, President of the Board of Trustees.

The Degree of M. D. was conferred on eight intelligent young men, as follows:

Archer Avery........................Georgia.
Thos. W. Gordon........................Georgia.
C. H. Jones........................Georgia.
J. B. Rutland........................Alabama.
J. W. Mitchell........................Georgia.
J. W. Bradley........................Georgia.
Thos. P. Davis........................Georgia.
M. M. Evans........................Alabama.

The *Ad Eundem* Degree was conferred on the following medical gentlemen, to-wit:

Alban S. Payne, M.D., of Virginia.
J. F. Alexander, M.D., of Georgia.
Wm. G. Owens, M.D , of Georgia.
A. T. Park, M.D , of Georgia.
T. E. Collier, M.D., of Georgia.
Irwin A. Cofer, M.D., of Georgia.
J. S. Todd, M.D., of Georgia.

The Address of President POWELL on the presentation of the Diplomas was one of rare beauty and eloquence, and replete with sound morals.

After the conferring of the Degrees, a card of thanks was read by the Dean addressed, by the students, to the Auxiliary Professors of the Institution. It was neatly and appropriately gotten up, and was warmly expressive of their gratitude to the Auxiliary members of the Faculty for the very efficient aid which they rendered to the excellent and varied course of instruction furnished by the Institution.

There was quite a profusion of boquets sent up to the graduates and students. A pleasing episode occurred in the presentation, by a lady, of a rich and luscious basket of fruit to Dr. J. W. Bradley and Dr. Thos. P. Davis, of the graduating class—Prof. OWENS delivering the gift to the happy recipients in his peculiar and pleasant style.

The Dean then returned thanks to the audience for their presence and polite attention, and the exercises were closed with the benediction by the Rev. Dr. HORNADY, pastor of the Third Baptist Church.

Upon the whole, the occasion was a most pleasant one, and well calculated to impress the observer with the high estimate placed upon this new enterprise by the citizens and by the Profession of Atlanta, and, in connection with the extraordinarily large class of sixty-four matriculates which attended its first session, gives promise of a bright and successful future for the Institution.

A catalogue containing a list of the students and full information relative to the advantages of the Institution will soon be issued, a copy of which will be cheerfully furnished to any one applying for it.

Address, R. C. WORD, M.D., Dean, Atlanta, Ga.

